# Declining Performance on American Board of Emergency Medicine Written Examinations

**DOI:** 10.1002/aet2.70105

**Published:** 2025-10-24

**Authors:** Earl J. Reisdorff, Samuel M. Keim, Diane L. Gorgas, Suzanne R. White, John L. Kendall, Kathleen C. Ruff, Felix K. Ankel, Susan E. Farrell, Yvette Calderon, Michael Gottlieb, Yachana Bhakta, Melissa A. Barton, Kevin B. Joldersma

**Affiliations:** ^1^ American Board of Emergency Medicine East Lansing Michigan USA; ^2^ Department of Emergency Medicine The University of Arizona College of Medicine Tucson Arizona USA; ^3^ Department of Emergency Medicine Ohio State College of Medicine Columbus Ohio USA; ^4^ Department of Emergency Medicine Wayne State University Detroit Michigan USA; ^5^ Department of Emergency Medicine Stanford School of Medicine, Stanford University Standford California USA; ^6^ Department of Emergency Medicine Regions Hospital St. Paul Minnesota USA; ^7^ Department of Emergency Medicine Brigham and Women's Hospital Boston Massachusetts USA; ^8^ Icahn School of Medicine, Mount Sinai Health System New York New York City USA; ^9^ Department of Emergency Medicine Rush University Medical Center Chicago Illinois USA

## Abstract

**Introduction:**

Emergency medicine (EM) is at a critical juncture with pervasive boarding and overcrowding, a rapid rise in new residency programs, and continuing recovery from the COVID‐19 pandemic. These factors could all potentially impact trainees' learning experiences. To explore how this has influenced trainee knowledge acquisition, we analyzed the trends in the American Board of Emergency Medicine (ABEM) In‐training Examination (ITE) and the written Qualifying Examination (QE).

**Methods:**

This was a retrospective study of multiyear performance trends for the ITE (2018–2024) and QE (2019–2024). Only ITE results from residents in categorical ACGME‐accredited EM programs were included. ITE performance was the aggregate mean scaled (equated) scores of all EM training levels. The measures for QE performance were the mean scaled scores (equated) and the pass rates. For each test, descriptive statistics were reported and an omnibus analysis of variance (ANOVA) comparing scores across years was computed. When an ANOVA result was statistically significant (*α* < 0.01), Tukey's tests were performed.

**Results:**

For the ITE, there were 61,512 test results, of which 59,075 (96.0%) met inclusion criteria. The mean (SD) scaled ITE scores declined from 77.36 (8.85) in 2018 to 72.19 (9.44) in 2024. The ANOVA for the ITE scaled scores was statistically significant (*p* < 0.01). The QE had 17,040 test results, of which 15,651 (91.8%) met inclusion criteria. The mean (SD) scaled scores declined from 82.8 (4.6) in 2019 to 80.5 (4.5) in 2024, while the pass rate also declined from 92.3% in 2019 to 82.0% in 2024. The ANOVA for the QE scaled scores across years was significant (*p* < 0.01).

**Conclusions:**

Physician performance on the ABEM ITE has steadily declined since 2018; performance on the QE has declined since 2019. Future research is needed to understand and address the potential causes of these trends.

## 1 Introduction

The breadth and depth of knowledge, skills, and abilities expected for an emergency physician to practice continues to expand as demonstrated by changes in The Model of Clinical Emergency Medicine over the past decade [1, 2]. A myriad of factors may adversely affect the emergency medicine (EM) resident's knowledge acquisition throughout residency. Systemic overcrowding and boarding of patients can decrease the number of new, undifferentiated patients seen by an EM resident [3, 4, 5, 6, 7, 8, 9, 10]. The COVID‐19 pandemic may have reduced exposure for trainees regarding both the number and breadth of cases, with lingering impacts on resident education still present [11, 12, 13, 14, 15, 16, 17]. Moreover, the rapid expansion in the number of EM residencies resulted in many new programs, which may have more limited educational infrastructure and lack the benefit of longstanding feedback and refinement afforded to more established programs [18].

The Accreditation Council for Graduate Medical Education (ACGME) emphasizes the importance of residency programs demonstrating the effective transfer of EM knowledge and skills to their graduates, with board certification serving as a key measure of this knowledge translation to protect the public [19]. However, the impact of these recent disruptions and changes to the training environment on residents' ability to acquire and demonstrate expertise in their knowledge of EM has not been systematically studied.

Two tests, the In‐training Examination (ITE) and the Qualifying Examination (QE), are established standards used by the field to evaluate knowledge of EM. The ITE is a formative assessment, primarily used by residency programs to evaluate preparedness to take the QE. The American Board of Emergency Medicine (ABEM) uses the QE as a summative assessment to determine whether a candidate has sufficient knowledge of EM to demonstrate sufficient knowledge for practice.

Identifying trends in ABEM ITE and QE performance during this period can provide valuable insights into whether these unique environmental changes may have interfered with residents' ability to learn the breadth and depth of EM. Understanding these trends is crucial for informing the specialty, providing direction for areas of future research, and improving the community's understanding about the relationship between the ITE and QE exam performance and the training environment. As the newest generation of emergency physicians prepare to enter practice, there is a critical need to understand how this combination of factors may have influenced the acquisition of specialty‐specific knowledge and board passage rates.

To address this gap, this study sought to analyze changes in performance on ABEM written examinations, the ITE and QE, during a period marked by substantial changes within the specialty and within healthcare delivery writ large.

## 2 Methods

This was a retrospective descriptive study that examined multi‐year performance trends for the ABEM ITE and QE. This study adheres to the Strengthening the Reporting of Observational Studies in Epidemiology (STROBE) guidelines [20]. The study was deemed exempt by the Western‐Copernicus Group (WCG) Institutional Review Board.

### 2.1 Test Descriptions and Metrics

The ITE is a secure 225 multiple‐choice item examination and is administered annually in late February or early March. The examination uses a secure platform, and data are stored on secure ABEM servers. ABEM reports the ITE as a scaled score (psychometrically equated using item response theory) ranging from 0 to 100. For this study, we used the mean scaled score as the measure of ITE performance, which was reported as the aggregate mean of all EM training levels. The ITE is constructed using a psychometric blueprint and statistically equated to ensure similar difficulty compared with prior examinations, making year‐to‐year and form‐to‐form comparisons valid.

The QE is a secure 305 multiple‐choice item examination and is administered in late fall annually. The QE is a summative examination that yields a pass‐fail decision. The examination uses secure professional testing centers, and performance data are transmitted to ABEM and stored on secure ABEM servers. The measure for QE performance was the mean score and the pass rate. QE pass rates were reported for first‐time test takers and for all test takers. The QE is constructed using a psychometric blueprint and is statistically equated to ensure similar difficulty compared with prior examinations, making year‐to‐year and form‐to‐form comparisons valid. ABEM also reports the QE as a scaled score (psychometrically equated using item‐response theory) ranging from 0 to 100.

### 2.2 Sample Windows

The ITE study period was from 2018 to 2024. This period was selected because prior to 2018, ABEM applied a different equating model, which limited comparability. Equating is a form of psychometric weighting that can compensate for variations in the year‐to‐year difficulty of a test. Equating ensures that residents are scored fairly and not disadvantaged for taking a more difficult exam. Prior to 2018, ABEM used an equipercentile equating method; for 2018 and later, ABEM used a common‐item equating method using the Rasch model [21].

The study period for the QE was from 2019 to 2024. In 2019, ABEM reset the passing standard for the QE. Passing standards are revisited periodically to ensure that the assessment is staying current with developments in a specialty. ABEM used a modified‐Angoff approach for the 2019 standard setting study [22]. Because we limited the study to tests that had a common passing standard and comparable score scale, QE results from 2018 and earlier were excluded.

We chose these limited time frames because adjustments in prior passing standards and equating techniques can affect scores and pass rates. ABEM typically resets assessment standards when there is a significant change in the Model of the Clinical Practice of Emergency Medicine [1]. The 2022 EM Model changes were determined to be insufficient to require repeat standard setting.

### 2.3 Sample Selection Criteria

Only ITE results from residents in categorical ACGME‐accredited EM programs were included. Results for residents in American Board of Medical Specialties‐International residencies were excluded, as were physicians in combined residencies and residents who were transferring into EM residencies from other specialties. For the QE, total test takers were reported, but analyses were based upon ABEM's reference group which is first‐time test takers who were residency trained.

### 2.4 Analyses

We present descriptive statistics by year, including mean and standard deviation (SD). An omnibus analysis of variance (ANOVA) comparing ITE scores across years was computed. For the QE, an omnibus ANOVA was also computed to compare mean scores across years, as well as pass rates across years. When an ANOVA result was statistically significant (*α* < 0.01 determined a priori), Tukey Honestly Significant Difference (HSD) tests were performed to determine which specific group means are significantly different from each other. Analyses were conducted using R version 4.4.0 (The R Foundation; Vienna Austria).

## 3 Results

**FIGURE 1:**
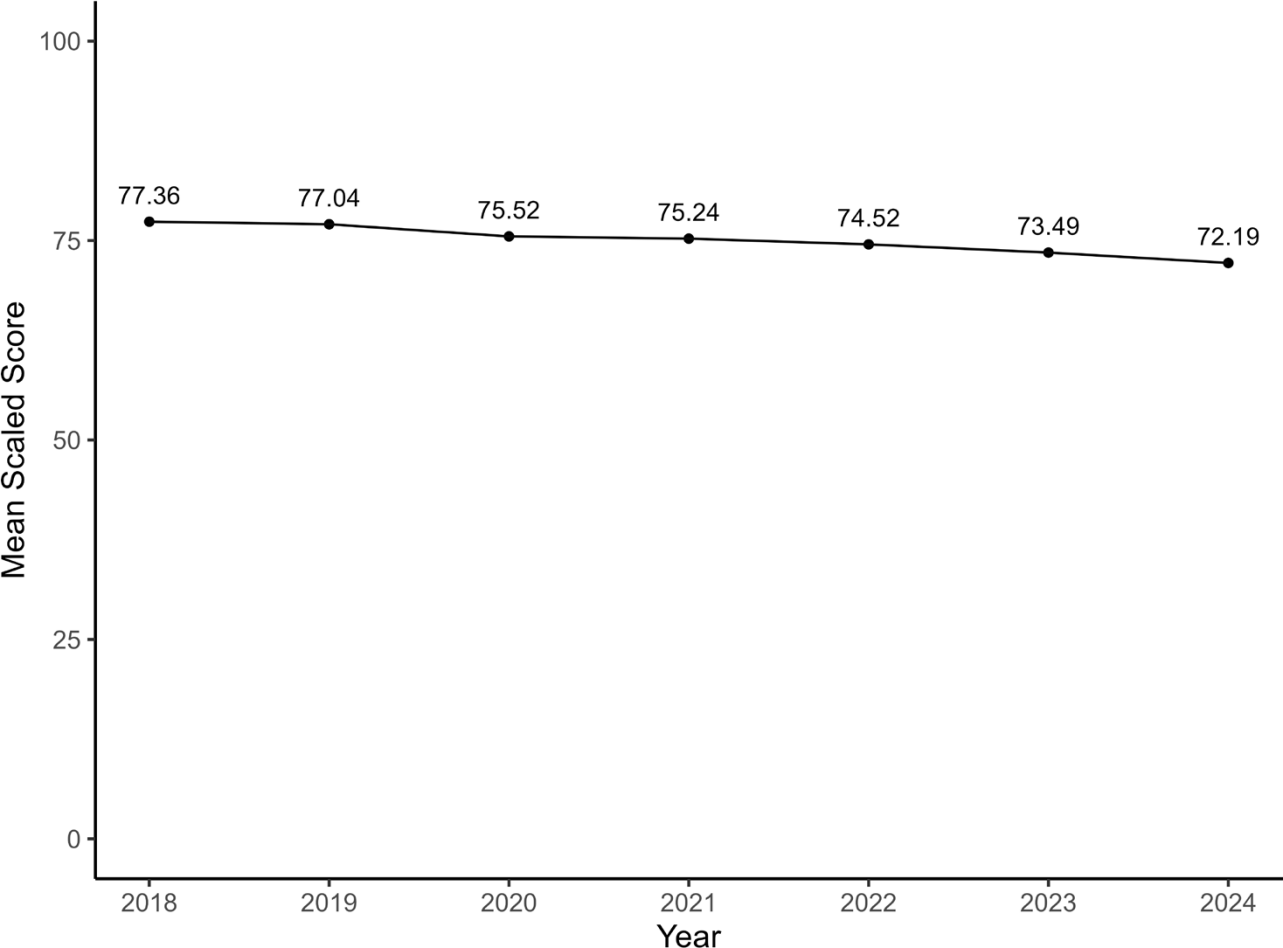
American Board of Emergency Medicine In‐Training Examination Mean Scaled Scores from 2018 to 2024 for categorical resident test takers.

**FIGURE 2:**
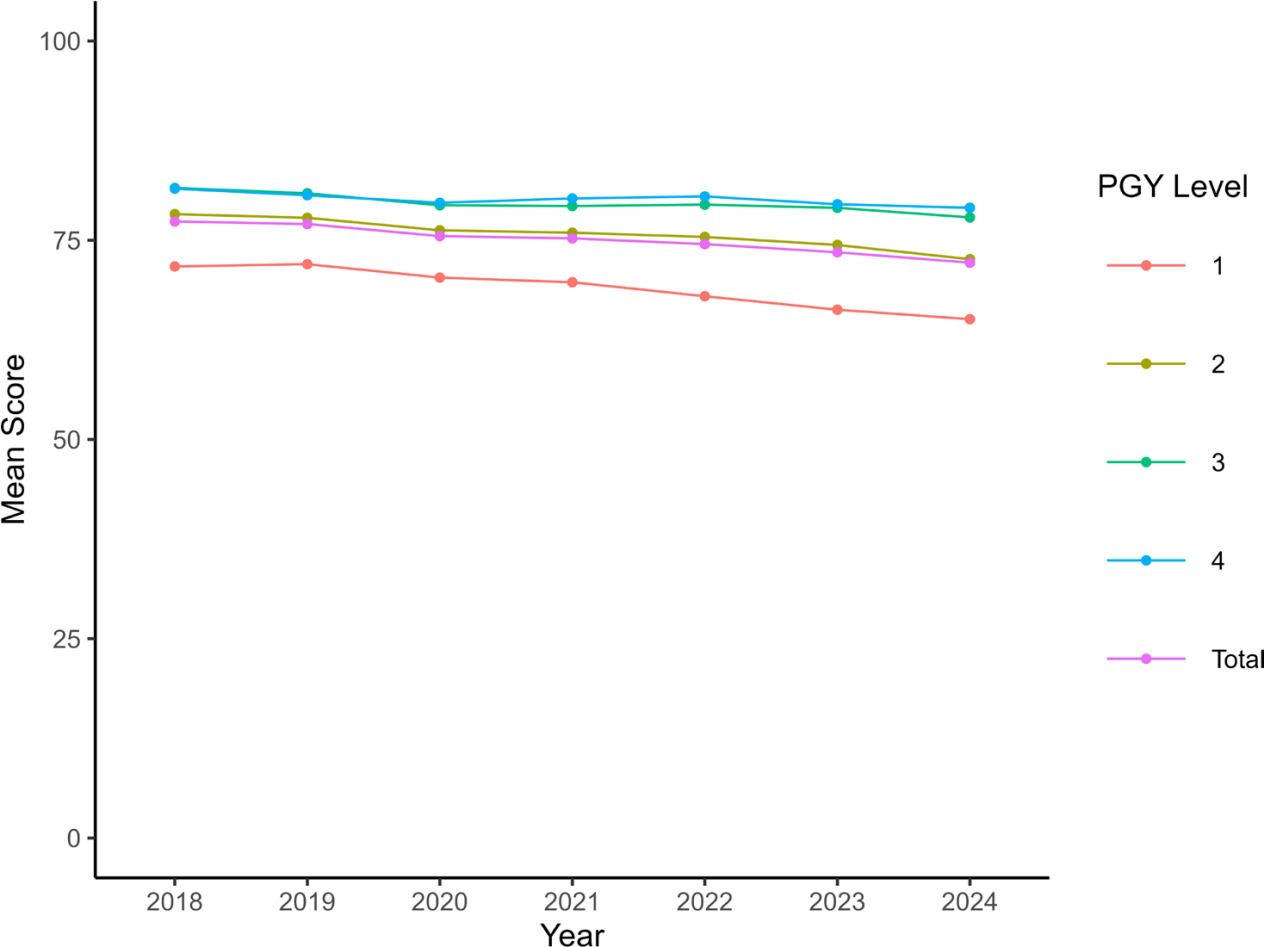
American Board of Emergency Medicine In‐Training Examination Mean Scaled Scores by level from 2018 to 2024 for categorical resident test takers. PGY, post‐graduate year.

For the ITE, there were 61,512 test results, of which 59,075 (96.0%) met inclusion criteria (Figures S1 and S2). The mean (SD) scaled scores demonstrated a progressive decline from 77.36 (8.85) in 2018 to 72.19 (9.44) in 2024 (Table 1). The ANOVA for the ITE scaled scores was statistically significant (*p* < 0.01; Figure 1). The Tukey's HSD tests were all significant except for the comparisons for 2019–2018 and for 2021–2020. This effect was notable across all post‐graduate levels (Figure 2, Table S1).

**TABLE 1:** American Board of Emergency Medicine In‐Training Examination Results 2018–2024 for categorical resident test takers.

Year	Included test takers	Mean (SD) scaled score
2018	7108	77.36 (8.85)
2019	7712	77.04 (7.82)
2020	8163	75.52 (8.23)
2021	8491	75.24 (8.39)
2022	8918	74.52 (9.17)
2023	9201	73.49 (9.30)
2024	9483	72.19 (9.44)

Abbreviation: SD, standard deviation.

**FIGURE 3:**
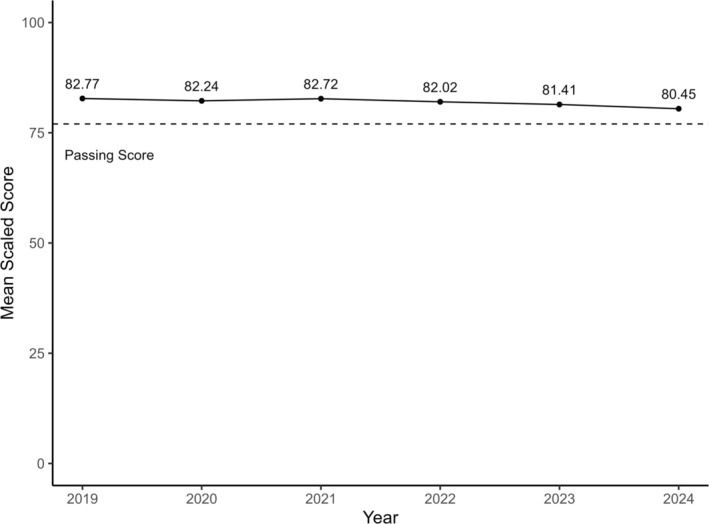
American Board of Emergency Medicine Qualifying Examination Mean Scaled Scores from 2019 to 2024 for test takers.

The QE had 17,040 test results, of which 15,651 (91.8%) met inclusion criteria. The mean (SD) scaled scores demonstrated a progressive decline from 82.8 (4.6) in 2019 to 80.5 (4.5) in 2024 (Table 2; Figure 3). The ANOVA for the QE scaled scores across years was significant (*p* < 0.01). Tukey's HSD tests were significant except for the 2021–2019 and 2022–2020 comparisons.

**TABLE 2:** American Board of Emergency Medicine Qualifying Examination Results 2019–2023.

Year	Total test takers, *n*	Reference group, *n*	Reference group passed, *n*	Reference group pass rate, %	Reference group fail rate, %	Reference group mean scaled score	Reference group standard deviation
2019	2496	2282	2107	92.3	7.7%	82.8	4.6
2020	2621	2426	2182	89.9	10.1%	82.2	4.7
2021	2685	2685	2424	90.3	9.7%	82.7	4.8
2022	2860	2608	2363	90.6	9.4%	82.0	4.5
2023	3027	2710	2387	88.1	11.9%	81.4	4.5
Total	13,689	12,711	11,463	—		—	—

Abbreviation: SD, standard deviation.

The pass rate also declined from 92.3% in 2019 to 82.0% in 2024. The 10.3% decline in the absolute pass rate of the QE was larger than the small change in mean scaled scores. This relationship—a small change in the scaled score resulting in a change of this magnitude in the pass rate—is seen when the change in mean scores is near the cut score (passing score). The ANOVA for pass rates was also significant (*p* < 0.01). Tukey's HSD tests were significant for every year compared to 2024. In addition, the Tukey HSD was also significant for 2023–2019 and 2023–2021.

## 4 Discussion

The field of EM faces a unique learning environment for trainees, with severe overcrowding and boarding issues, the COVID‐19 pandemic, and rapid increases in new programs, with limited understanding of the broadscale impact of these factors across current trainees. This investigation presents important longitudinal performance data on written ABEM examinations administered amidst these significant transitions.

In this study, we found a progressive decline in ITE scores, QE scaled scores, and QE pass rates from 2018–2019 to 2024. While the specific score changes may be small in magnitude when compared year to year, this study provides evidence of an important signal which bears attention in our specialty. Moreover, despite small absolute differences in QE scores, these differences can have large impacts on pass rates when mean scores are close to the minimum passing score because the mean would typically be near the thickest part of the distribution of candidate scores. A related and important psychometric concept is the relative precision of the QE scores, known as the Standard Error of Measurement (SEM). The average value of the SEM over the study window was 1.88. Given that the average score is nearly three SEMs away from the average score, it is unlikely that score precision would explain the decline in scores.

The literature is sparse with regard to factors associated with performance on the ABEM ITE. There is evidence from other specialties that may inform this study and serve as an area of future research for EM. There was a positive association between residency program size, duration, and faculty complement and performance on the American Board of Surgery Certification Examination [23]. This study also found that more mature programs and programs with higher percentages of residents graduating from United States (US) allopathic programs compared with international or osteopathic schools were associated with improved performances [23]. In a study of the American Board of Pediatrics (ABP) written certifying examination (CE), the following three residency factors were associated with better performance: ratio of full‐time paid faculty to residency positions; percentage of US medical graduates; and time spent in regularly scheduled lectures [24]. In EM, there has been a rapid rise in the number of ACGME‐accredited EM residencies, increasing from 220 programs in 2018 to 292 programs in 2024 [25, 26, 27, 28]. Moreover, from 2015 to 2024 there were 47 categorical residencies that transitioned from American Osteopathic Association (AOA)‐approved to ACGME accreditation under the Single Accreditation System. The rapid expansion of the number of EM residencies could have added some programs with less exposure to complex medical conditions or simply lack the longevity to have established a successful educational curriculum. It is unclear as to the impact that the expansion in the number of EM residencies has contributed to the decline in performance on the ITE and QE but, given that newer programs may have fewer resources and infrastructure, this warrants further exploration [29].

Moreover, there were notable changes with regard to EM residency training during the study period. The EM training community was faced with a reduction in core faculty dedicated time, which may have reduced the available educational support and quality of didactics for learners [30, 31, 32]. The ACGME Milestones, markers to demonstrate the acquisition of knowledge, skills, and abilities along the continuum of residency training, were also updated [33]. If the curricula designed by faculty were influenced by changes to the Milestones, it is possible that some residents may not have received training in line with those new learning objectives thereby leading to potential knowledge gaps due to reliance on older curricula.

The characteristics of students applying and matching into EM also changed over time. In 2017, there were 2047 EM‐1 resident positions offered in the 2017 NRMP Match, of which approximately 78.2% were filled by US doctorate of medicine (MD) seniors, 13.8% US doctorate of osteopathy (DO) seniors, 4.2% US international medical graduates (IMGs), and < 1% non‐US IMGs [34]. By comparison, in 2025, there were 3068 EM‐1 resident positions offered, of which approximately 44.9% were filled by US MD seniors, 35.1% US DO seniors, 10.3% US IMGs, and 4.3% non‐US IMGs [35]. It is conceivable that the increasing availability of EM residency positions may have changed the applicant pool by comparison to previous years. For example, in 2024, there was less than one applicant (0.98) per available EM resident position and 98% of US MD seniors matched into EM as their first choice [36]. The shift in the distribution of medical school training may also have influenced the preparation and baseline knowledge entering residency, thus influencing their resulting ITE and QE scores. A similar finding has been seen in literature from other specialties demonstrating an association between medical school training and CE performance [37].

However, there are likewise numerous environmental factors that have changed within healthcare delivery over this time beyond simply the characteristics of the applicant and programs. For example, the COVID‐19 pandemic had a substantial impact on medical education. In addition to impacts on mental health and social isolationism with its downstream ability to learn, there was a decrease in face time with educators, a decrease in case volume and variety during the pandemic, and an overall disruption of educational opportunities [38, 39, 40, 41]. However, the downward trend in ITE performance was noted prior to the onset of the pandemic and continued after the crisis phase of the pandemic was over. The same pattern is suggested, albeit less demonstrably, in the QE results. For the QE, the pass rate decreased from 92.3% to 88.1%. The impact of this difference on the 2023 QE is 323 first‐time test takers failing the QE (the actual 88.1% pass rate) compared with 209 first‐time test takers failing the 2019 QE (if the 92.3% pass rate from 2019 was applied). Because the downward trend was noted prior to the pandemic, it is unlikely that COVID‐19 was the sole factor, but may have been a contributing factor to the decline in scores.

The prevalence of burnout among resident and attending emergency physicians is another substantial factor that influences emergency care [41]. Burnout also poses a risk to the learning experience and educational retention. Burnout has been rising and there was a substantial increase in the incidence of burnout during the COVID‐19 pandemic [3, 42] Despite this, no clear associations between resident burnout and ITE performance have been found [42, 43].

Patient boarding is another factor that may influence the learning experience by reducing access to new and diverse cases. Recent research has shown that boarding and overcrowding, as well as the expansion of the provider‐in‐triage role can negatively impact trainee experiences [44]. As boarding continues to rise, this may play a role in the decline in scores and demonstrate yet another harm due to boarding and overcrowding [45]. While a full discussion of boarding is beyond the scope of this paper, educators should consider using this time to optimize trainee education with boarding‐specific educational initiatives [46, 47].

Another possible explanation for the observed trend would be whether the assessments themselves are stable metrics to draw effective comparisons. To this end, all ABEM examinations adhere to standard psychometric practices for question development [48]. Each examination is constructed and statistically equated to ensure similar difficulty compared with prior examinations; thus, a decline in performance over time most likely reflects differences in the test takers medical knowledge and cognitive skills, rather than the psychometric characteristics (e.g., difficulty) of the examination per se. Consequently, it is highly unlikely that the trend could be explained by measurement error or psychometric variation of the assessments.

Notably, the decline in ITE scores was consistent across all post‐graduate years with the most pronounced declines seen among first‐ and second‐year residents. This may reflect a more proximate effect to these environmental factors. However, the extension of this decline to senior‐level residents suggests that this extends beyond only initial training with a sustained effect throughout their training.

The downward trend in ITE performance suggests that a continued decline in QE performance could be expected. The nadir of the QE performance may be yet to come if there is not a self‐correction (i.e., increased exam preparation, greater attention to knowledge gaps) to some degree. If the performance trends on ABEM written examinations continue, the specialty should closely examine the educational experience of residency. ABEM will also continue to reevaluate the validity of its existing and future assessment processes [49]. Future research to establish the relationship between resident knowledge and these environmental factors, such as burnout, faculty ratios, or program quantity and quality, is needed. This could include focused questions associated with the ITE or QE to identify these factors, as well as surveys of programs and other environmental factors.

## 5 Limitations

This study had several limitations. First, it was a retrospective observational study and could not identify the degree to which purported factors influenced examination performance. This time period aligned with multiple program and environmental changes that may have impacted the findings. Future work is needed to elucidate the degree to which each element may have impacted these scores. Second, we did not examine any performance differences in the residency format (EM1‐3 versus EM1‐4). However, prior analyses showed that test performances on the QE were similar, with slightly higher performances for physicians from EM1‐3 programs [49]. Given the stable ratio of EM1‐3 to EM1‐4 programs during the study periods, the impact of training duration, if any, is likely to be minimal. Third, we limited this study to ABEM's written examinations. The COVID‐19 pandemic had a dramatic effect on the administration of the ABEM oral exam (e.g., temporarily halting the oral exam administration), and so we opted to exclude those results from our analysis. Interpreting these trends should be approached cautiously because the time windows are short. Finally, changes in the examination assessment prior to 2018–2019 limited the comparability for this study and continued work will be needed to assess this over future time periods to identify the persistence of this trend or when a nadir may occur.

## 6 Conclusions

Physician performance on the ABEM ITE has been declining since 2018; performance on the QE has declined since 2019. The factors contributing to these trends are uncertain. Future studies are needed to better understand and address the potential causes for these trends.

## Author Contributions

Study concept and design: E.J.R., M.G., K.B.J. Acquisition of the data: K.B.J. Analysis and interpretation of the data: M.G., K.B.J. Drafting the manuscript: E.J.R., S.M.K., D.L.G., J.L.K., S.R.W., M.G., M.A.B., Y.B. Critical revision of the manuscript: E.J.R., S.M.K., D.L.G., J.L.K., S.R.W., K.C.R., F.K.A., S.E.F., Y.C., M.G., M.A.B. Statistical expertise: K.B.J.

## Conflicts of Interest

Dr. Joldersma, Dr. Gottlieb, Dr. Barton, Ms. Ruff, and Ms. Bhakta are employees of the American Board of Emergency Medicine (ABEM). Dr. Reisdorff was previously employed by ABEM. Drs. Gorgas, White, Kendall, Ankel, Farrell, and Calderon serve on the ABEM Board of Directors. Dr. Keim served on the ABEM Board of Directors during the study period. ABEM receives revenue from the In‐training Examination, the Qualifying Examination, and the Oral Certifying Examination.

## Supporting information


**Data S1:** Supporting Information.

## Data Availability

The data that support the findings of this study are available on request from the corresponding author. The data are not publicly available due to privacy or ethical restrictions.
