# Supplementary material for: Declining Performance on American Board of Emergency Medicine Written Examinations
Source: AEM Educ Train. 2025 Oct 24;9(5):e70105. doi: 10.1002/aet2.70105 (PMC12552112; doi:10.1002/aet2.70105)
Supplement: Supplementary file 1 — Data S1: Supporting Information. [file AET2-9-e70105-s001.docx]

**Supplemental Figure 1. Flow Diagram for In-training Examination Analysis**

Assessed for eligibility (n = 61,512)

Analyzed (n = 59,075)

Excluded as resident from a non-categorical (PGY1-3 or PGY 1-4) training program (n = 2,437)

**Supplemental Figure 2. Flow Diagram for Qualifying Examination Analysis**

Assessed for eligibility (n = 17,040)

Analyzed (n = 15,651)

Excluded multiple takers (n = 1,389)

**Supplemental Table 1. American Board of Emergency Medicine In-training Examination Results 2018-2024 for Categorical Resident Test Takers by Post-Graduate Year.**

| Level | Year | Included Test Takers | Mean (SD) Scaled Score |
| --- | --- | --- | --- |
| 1 | 2018 | 2,308 | 71.72 (7.80) |
|  | 2019 | 2,501 | 72.00 (6.94) |
|  | 2020 | 2,633 | 70.31 (7.41) |
|  | 2021 | 2,751 | 69.73 (7.29) |
|  | 2022 | 2,927 | 67.98 (7.80) |
|  | 2023 | 2,983 | 66.29 (7.65) |
|  | 2024 | 3,067 | 65.10 (7.74) |
| 2 | 2018 | 2,171 | 78.28 (7.89) |
|  | 2019 | 2,389 | 77.82 (7.03) |
|  | 2020 | 2,503 | 76.25 (7.12) |
|  | 2021 | 2,626 | 75.95 (7.46) |
|  | 2022 | 2,746 | 75.41 (7.79) |
|  | 2023 | 2,894 | 74.41 (7.63) |
|  | 2024 | 2,940 | 72.62 (7.77) |
| 3 | 2018 | 2,037 | 81.56 (7.73) |
|  | 2019 | 2,206 | 80.90 (6.64) |
|  | 2020 | 2,392 | 79.39 (7.19) |
|  | 2021 | 2,477 | 79.30 (7.08) |
|  | 2022 | 2,620 | 79.47 (7.73) |
|  | 2023 | 2,701 | 79.07 (7.41) |
|  | 2024 | 2,869 | 77.87 (7.61) |
| 4 | 2018 | 592 | 81.48 (8.01) |
|  | 2019 | 616 | 80.65 (6.98) |
|  | 2020 | 635 | 79.70 (7.70) |
|  | 2021 | 637 | 80.25 (7.87) |
|  | 2022 | 625 | 80.50 (7.54) |
|  | 2023 | 623 | 79.52 (7.95) |
|  | 2024 | 607 | 79.08 (8.25) |

*SD, standard deviation*
